# Geometrical model for calculating the effect of surface morphology on total x‐ray output of medical x‐ray tubes

**DOI:** 10.1002/mp.14649

**Published:** 2021-02-17

**Authors:** Maximilian Siller, Mika Minkkinen, Pamela Bogust, Alexander Jelinek, Jürgen Schatte, Neil Bostrom, Kasey Greenland, Wolfram Knabl, Helmut Clemens, Reinhard Pippan, Verena Maier‐Kiener

**Affiliations:** ^1^ Department of Materials Science Montanuniversität Leoben Franz‐Josef‐Straße 18 Leoben 8700 Austria; ^2^ Varex Imaging Corp 1678 Pioneer Rd Salt Lake City UT 84104 USA; ^3^ Erich Schmid Institute of Materials Science Austrian Academy of Sciences Jahnstraße 12 Leoben 8700 Austria; ^4^ Plansee SE Metallwerk‐Plansee‐Straße 71 Reutte 6600 Austria

**Keywords:** laser scanning confocal microscopy, rotating anode, surface damage, surface roughening, tube aging, tungsten, x‐ray tube

## Abstract

**Purpose:**

Correlation of characteristic surface appearance and surface roughness with measured air kerma (kinetic energy released in air) reduction of tungsten‐rhenium (WRe) stationary anode surfaces.

**Methods:**

A stationary anode test system was developed and used to alter nine initially ground sample surfaces through thermal cycling at high temperatures. A geometrical model based on high resolution surface data was implemented to correlate the measured reduction of the air kerma rate with the changing surface appearance of the samples. In addition to the nine thermally cycled samples, three samples received synthetic surface structuring to prove the applicability of the model to nonconventional surface alterations. Representative surface data and surface roughness values were acquired by laser scanning confocal microscopy.

**Results:**

After thermal cycling in the stationary anode test system, the samples showed surface features comparable to rotating anodes after long‐time operation. The established model enables the appearance of characteristic surface features like crack networks, pitting, and local melting to be linked to the local x‐ray output at 100 kV tube voltage ,10° anode take off angle and 2 mm of added Al filtration. The results from the conducted air kerma measurements were compared to the predicted total x‐ray output reduction from the geometrical model and show, on average, less than 10 % error within the 12 tested samples. In certain boundaries, the calculated surface roughness R_a_ showed a linear correlation with the measured air kerma reduction when samples were having comparable damaging characteristics and similar operation parameters. The orientation of the surface features had a strong impact on the measured air kerma rate which was shown by testing synthetically structured surfaces.

**Conclusions:**

The geometrical model used herein considers and describes the effect of individual surface features on the x‐ray output. In close boundaries arithmetic surface roughness R_a_ was found to be a useful characteristic value on estimating the effect of surface damage on total x‐ray output.

## 1 INTRODUCTION

**Fig. 1:**
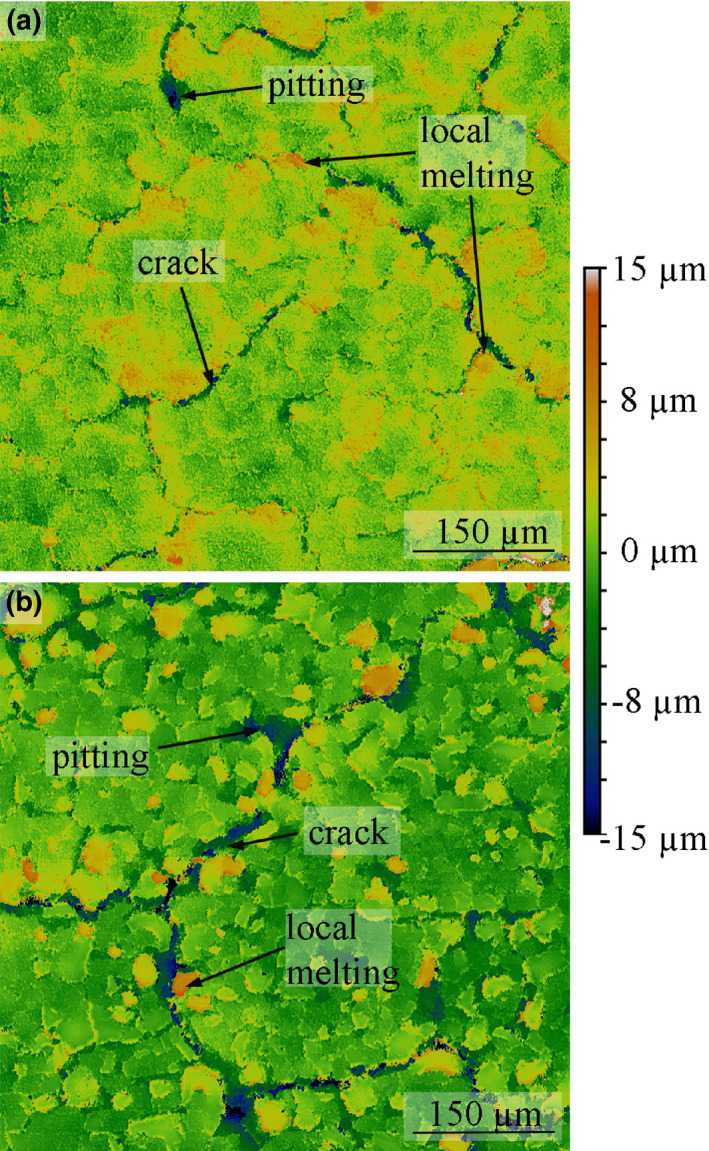
(a) Characteristic features like crack networks, melt droplets and pitting can be found on the surface of used rotating anodes; (b) in heavily damaged areas melt droplets become more frequent — laser scanning confocal microscopy images.

**Fig. 2:**
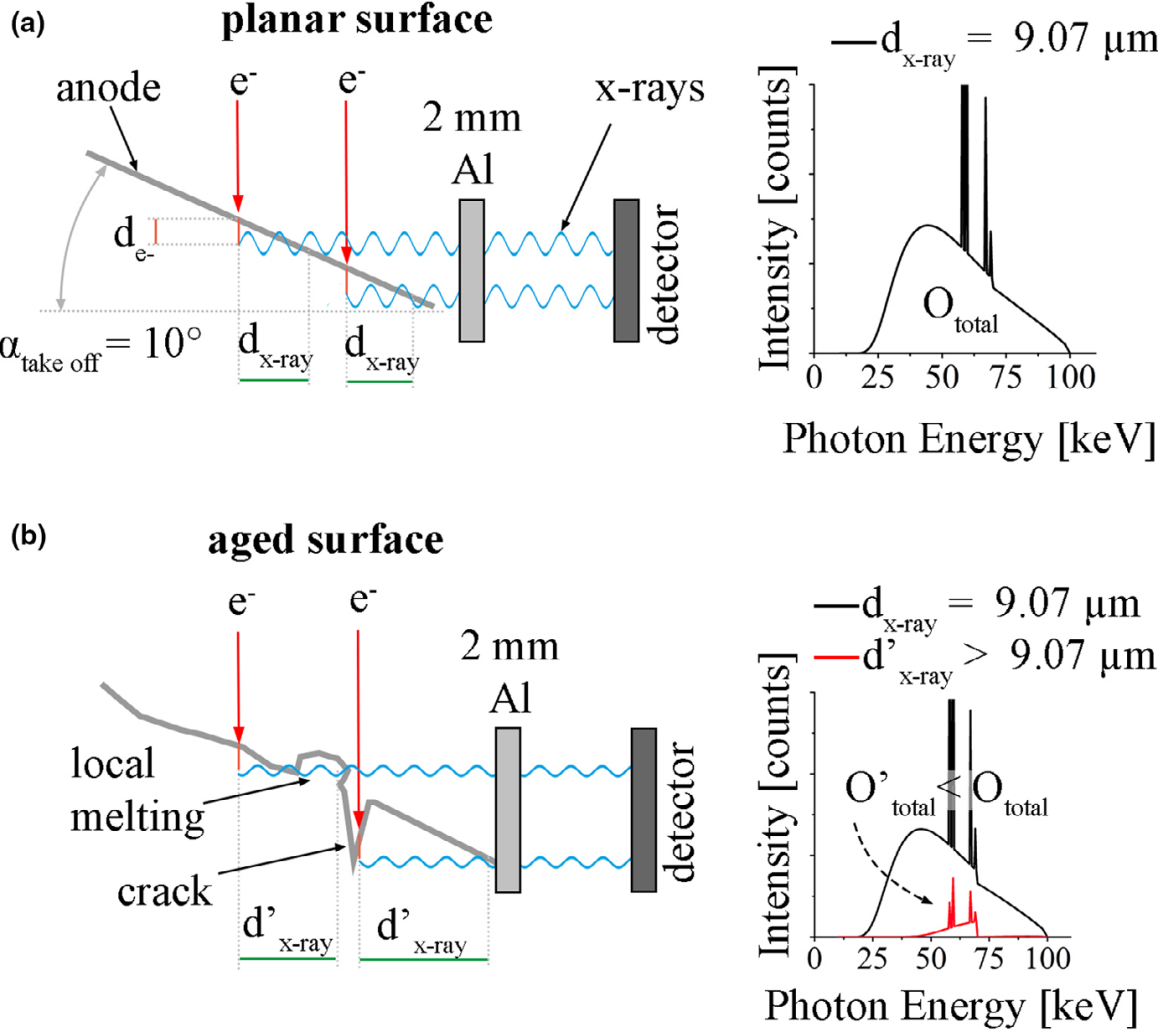
(a) A planar anode surface with constant d_x‐ray_ is leading to constant local x‐ray output, O_total_, along the surface; (b) formation of cracks or melt droplets leads to locally increased internal filtration d’_x‐ray_ and in turn to a reduced total x‐ray output (O’_total_).

During their long‐time service in medical imaging, the emitted x‐ray output of high‐performance x‐ray tubes may decrease depending on the operational parameters. This process is called tube aging and is mainly caused by the degradation of the anode surface.^1^, ^2^, ^3^, ^4^ Especially in computed tomography (CT), tube aging can lead to photon starvation during image acquisition and reduces the diagnostic value of the acquired data. Inside an x‐ray tube, electrons which are originating from the cathode, are accelerated toward the anode surface to generate x rays on the impact. The major drawback of this method is that a large quantity of the applied electrical power will be converted into heat in the focal spot. In order to prevent the metallic surface in the focal spot from melting and evaporating in a matter of microseconds, the anode is rotated below the incoming electrons. The heating power is consequently distributed over a larger area with an annulus‐like shape, the focal track. Every time the material passes through the incoming electron beam, the surface will experience a steep temperature rise and immense thermally induced stresses.^5^, ^6^ The surface can get damaged through cracking during this process if the stresses are high enough. Figure 1(a) shows the characteristic surface appearance at an intermediate level of surface damage using laser scanning confocal microscopy (LSCM). Besides cracking, other mechanisms of surface degradation such as pitting and local melting, Fig. 1(b), can be observed frequently in aged rotating anodes. Both effects are caused by cracks branching below the surface and isolating local areas from its surroundings.^7^, ^8^ W has excellent properties as a track material of an x‐ray tube through its combination of high thermal conductivity, high melting point, low vapor pressure, high volumetric heat capacity, and high atomic number.^9^ For further improvement of performance 5–15 wt.% Re (WRe5‐WRe15) can be added to reduce the formation of cracks and surface degradation.^10^ The first schematic model for the general impact of a damaged anode surface on the total x‐ray output was reported by Sedlatschek and Elsas.^10^ The decrease in output is caused by locally increased absorption due to the modification of the surface, which creates an increase in internal path length for photons.^1^, ^2^, ^3^, ^4^, ^5^, ^7^, ^11^ When accelerated electrons hit the surface of the W anode, only a small portion of the incoming electrical power, at normal conditions around 1%, will generate x rays. Literature values for the average penetration depth of electrons, d_e_‐, in W at an acceleration voltage of 100 keV range from 1 µm to 1.6 µm ^4^, ^12^. In Fig. 2(a) x rays, generated below a planar surface, will have to travel through a path of material, d_x‐ray_, and the applied filtration to reach the detector. The filtration in Fig. 2 consists of 2 mm Al. For a planar surface the value of d_x‐ray_ is independent of the position on the surface and calculated through the simple geometrical relation:

(1) $$d_{x‐ray}=\frac{d_{e‐}}{\text{tan}\left(\alpha_{\mathrm{takeoff}}\right)},$$

with α_take off_, representing the take‐off angle. In computed tomography applications α_take off_ is frequently set to 10° which, with d_e_‐ = 1.6 µm, results in d_x‐ray_ = 9.07 µm. Figure 2(b) visualizes the case of a damaged anode surface. In this case d’_x‐ray_(y) is increased locally due to surface alterations like cracks, pitting, or local melting which decrease the total x‐ray output (O_total_). Various authors have successfully modeled the spectra of planar anode surfaces which can be used to calculate the expected reduced total x‐ray output O’_total_ resulting from d’_x‐ray_(y).^13^, ^14^, ^15^, ^16^, ^17^, ^18^, ^19^, ^20^, ^21^

In contrast to earlier publications, in which the surface roughening was also successfully linked with measurements or simulations, this work uses high‐resolution surface data that accurately depicts the surface morphology of modern high‐performance x‐ray anodes.^1^, ^3^, ^5^, ^7^ In addition to the changes in the internal filtration due to surface roughening investigated in the past, the extensive shading effects of large surface features can be made visible. The latter turned out to be decisive for the calculation of the x‐ray output, which showed a good agreement with the air kerma data of the stationary anode test system.

Stationary anodes were chosen due to lower cost, better availability of materials, and higher reproducibility of results when compared to rotating anodes. Thermal cycling the stationary anodes by pulsed electron beam loading generated the characteristic surface appearance of aged rotating anodes. It was investigated which surface features should be avoided when aiming for high x‐ray output and how orientated surface structuring does influence the total x‐ray output.

## 2 MATERIALS AND METHODS

### 2.A Materials

A variety of different W based alloys, which differed mainly in microstructure and surface treatment, were investigated in the scope of this work. All samples consisted of a nominal 1 mm thick WRe10 surface layer applied on a particle strengthened Mo base substrate, MHC. The Re content of the 1 mm thick surface layer was in the range of 9–10 w.% and was verified by atom emission spectroscopy using an Icap 6500 DUO. The general shape of the samples can be described as cylindrical with a diameter of 38 mm and a height of 15 mm. α_take off_ in relation to the detector was 10°. The samples were manufactured from rotating anode preproducts or aged rotating anodes from Plansee SE with electric discharge machining and conventional machining. Nine samples, sample A to sample I, initially had a fine ground surface finish with a surface roughness R_a_ of about 0.6 µm.

The samples J, K, and L received surface structuring before any testing was done. The surface structuring was realized by femto second laser machining and wire discharge machining. The pulse duration of the femto second laser was 500 fs and the scan speed was 5 mm/s. The fluency was 1 J/cm^2^ and the pulse frequency was set to 10 kHz. For more details on the used system see Ref. [22]. A 254 µm wire diameter was used for the wire discharge machining. Afterwards the samples were cleaned mechanically and chemically for ultra‐high vacuum standard. All samples received a final heat treatment of 1350 °C for several hours in H_2_ atmosphere.

### 2.B Stationary anode test system and air kerma measurement

**Fig. 3:**
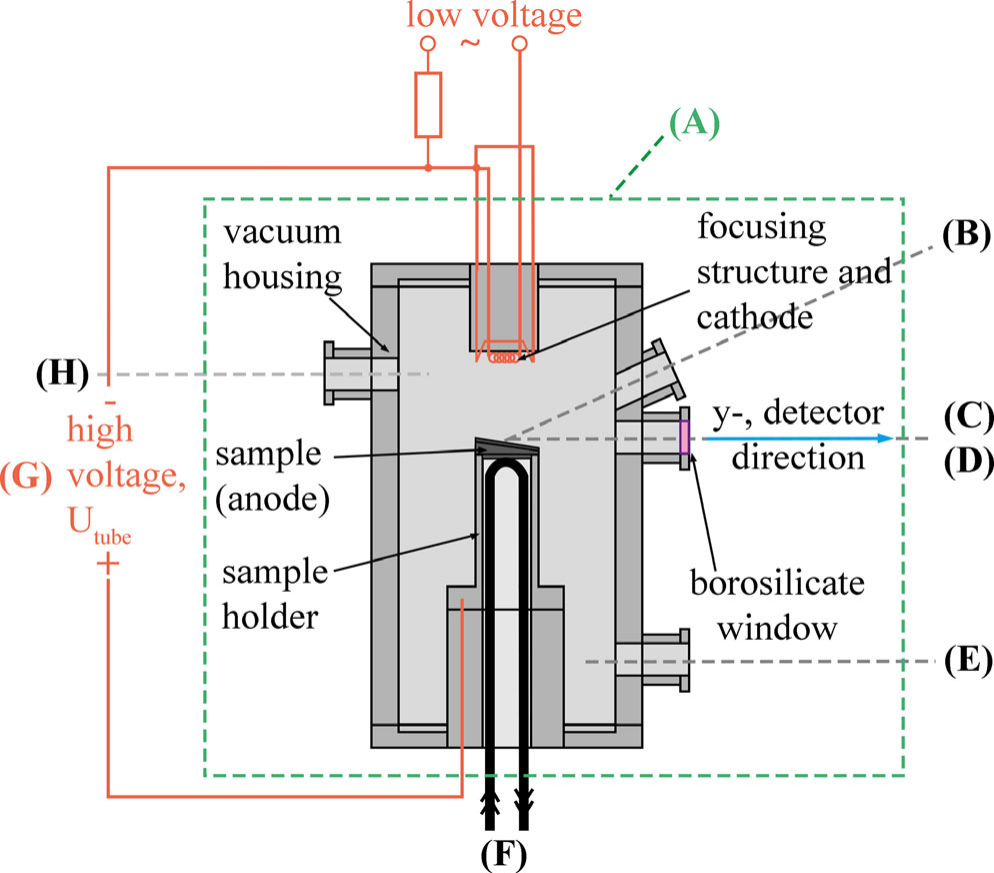
Schematic view of the main testing unit (a) and the attached analytic and operational devices (see text).

For damaging the sample surface and measuring the air kerma rate afterwards, a novel test system was designed. The test system, Fig. 3, includes the following devices:

- main testing unit (MTU)
- pyrometer
- x‐ray multimeter
- image sensor
- high vacuum system
- cooling system
- x‐ray generator
- hot‐filament ionization gauge

The main testing unit contains the sample and largely resembles a stationary anode x‐ray tube. During the exposures, the coolant flow was held constant to ensure a consistent cooling capacity. For measuring the focal spot temperature, a pyrometer was attached on one of the ports. The pyrometer was calibrated using known temperatures of W emitters. The emissivity and transmissivity values were set to 0.35 and 0.9, respectively. The electron intensity distribution in the focal spot was acquired through the measurement of the local x‐ray emission intensity with an image sensor. Later the analog image was processed with LabVIEW 2017 into an electron intensity distribution. The air kerma rate measurements were conducted with the distance between the x‐ray multimeter (detector) and the sample set to 150 mm. The angle between the sample surface and the detector was 10°. Between the detector and the sample surface, the x rays had to travel through a borosilicate glass window with 2.5 mm thickness and, if applied, additional 2 mm Al filtration. Figure 3 also shows a schematic visualization of the individual parts of the MTU. The samples were brazed on the sample holder which was attached on the bottom of the anode side of the MTU.

**Fig. 4:**
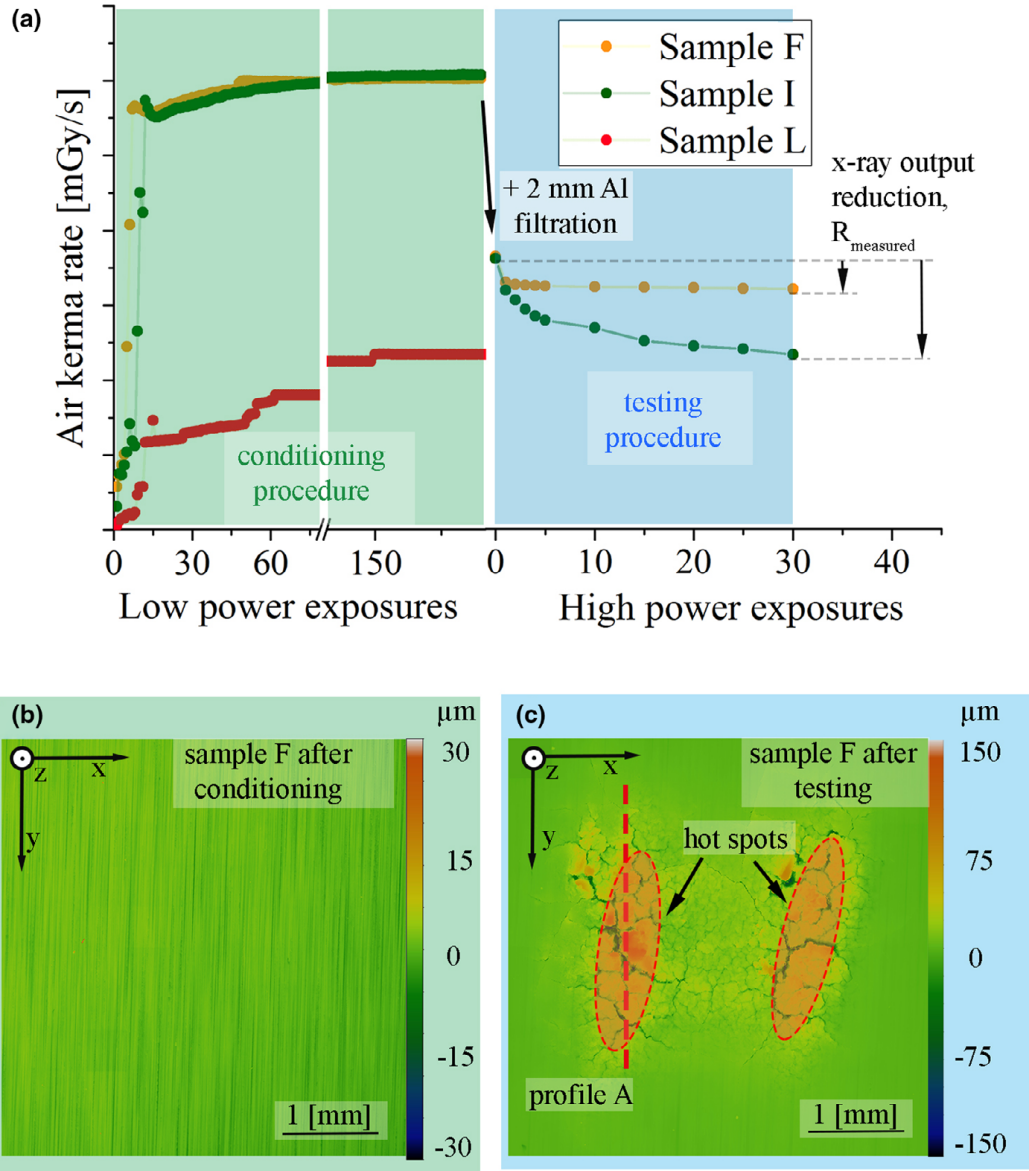
(a) Measured air kerma rate during conditioning and testing procedure. The schematic evaluation of R_measured_ (see text) is shown by the black arrows on the right hand side. Representative sample surface appearance after the conditioning (b) and the testing procedure (c) — laser scanning confocal microscopy images.

Proper precaution and vacuum practices were followed after venting the system and before starting any testing. The pressure inside the chamber before starting the conditioning was below 1 × 10^‐6^ Pa. Before being able to measure the initial air kerma level of any new sample, the MTU had to be conditioned by running a large amount of low power exposures. This proved to be necessary since unconditioned samples turned out to be susceptible to high voltage arcing and inconsistent air kerma data. The goal of the conditioning was to heat up, clean, and stabilize the system performance, without altering the target surface. During conditioning, the pressure in the MTU first increased, caused by the temperature rise inside the MTU during the exposures, and then decreased to around 3 × 10^−7^ Pa due to the active vacuum system. Figure 4(a) shows schematically the conditioning and test procedure. After 150 low power exposures the measured air kerma rate stabilized for sample F and I. One exposure is defined by the tube current I_tube_, the tube voltage U_tube_, the exposure frequency f_exp_, the shot time t_shot,_ and the exposure time t_exp_. During the conditioning procedure both U_tube_ and I_tube_ were increased steadily, starting from 50 kV and 10 mA up to 100 kV and 40 mA. The parameter t_shot_, f_exp_, and t_exp_, were set to 5 ms, 50 s^‐1^, and 30 s, respectively, consequently resulting in 1500 individual shots per exposure. The highest and last loading schedule during the conditioning procedure, called the conditioning schedule, U_tube_ = 100 kV and I_tube_ = 40 mA, leads to a maximum surface temperature of about 900 °C. After conditioning, the measured air kerma rate of all nine standard samples, sample A to I, which started with a ground surface, differed <4%. The samples possessed no measurable surface alteration after running the conditioning schedule multiple times as visualized in Fig. 4(b). After the conditioning procedure 2 mm Al filtration was added.

During the testing procedure, the samples were damaged through applying 30 high power exposures at increased U_tube_ and I_tube_ with reduced t_shot_ values. The maximum values for U_tube_, I_tube,_ and t_shot_ were 120 kV, 145 mA, and 2 ms, respectively, which resulted in a maximum temperature of 2300 °C and a temperature rise of 1000 °C at each shot. The initial x‐ray output was measured and compared with the value after testing to obtain the reduction in output. The applied U_tube_ and I_tube_ were varied between the different samples to generate different levels of surface damage. As can be seen in Fig. 4(a) between the high‐power exposures the samples were loaded with the conditioning schedule in order to measure the resulting drop of air kerma rate but not damage the surface further during the measurement. The intermediate conditioning schedules were applied five times after one to five high power exposures and then averaged. Finally, the measured air kerma rate after 30 exposures was compared with the starting values when 2 mm Al was added and calculated to the measured total x‐ray output reduction (R_measured_) for each sample. It should be pointed out that the targets, which were damaged during the testing procedure at the MTU with this approach, usually had a very characteristic surface appearance as shown in Fig. 4(c). The two areas which exhibit higher amounts of surface damage are resulting from local differences in electron intensity and are marked as hot spots in Fig. 4(c). In addition to the above test approach, sample J to L with their prealtered surfaces, were investigated by only comparing their air kerma rate at the end of the conditioning procedure. The R_measured_ for these samples was calculated by comparing the air kerma rate at the end of the conditioning procedure with the averaged air kerma rate of samples A to I at the same stage of processing.

### 2.C Surface measurement

For acquiring the LSCM images an Olympus LEXT OLS4100 was used. The x‐ and y‐resolution of the height maps, M_height_(x,y), was 5090 × 5090 pixels. Afterwards, the dataset was postprocessed with the software Gwyddion 2.48 (http://gwyddion.net/) for leveling, spike‐ and outliner‐removal. The zero plane of the dataset was set to the initial surface. The arithmetic surface roughness of the samples, R_a_, was calculated by averaging the calculated R_a_ values for 1000 profiles in the y‐direction, the direction toward the detector, for each sample. The area of the R_a_ calculation was 4 × 4 mm^2^ with the focal spot in the center.

### 2.D Software

The M_height_(x,y) was imported to Matlab R2017b 64bit (mathworks.com) for further data handling. For calculating the total x‐ray output reduction resulting from additional local filtration, SpekCalc pro 1.1 (http://spekcalc.weebly.com/) was used.^12^, ^16^ The total x‐ray output was calculated with the base filtration setup which consisted of 2.5 mm borosilicate glass and 2 mm Al as visualized in Fig. 3 and then was compared with the x‐ray output with the additional internal WRe10 filtration. Since W and Re differ only minimal in atomic number and density, the filtration characteristics of WRe10 are practically identical to pure W.^2^, ^12^ The resulting relation between additional internal W/WRe10 filtration and the x‐ray output was fitted with a double exponential decay function, f_red_(x).

## 3 RESULTS

### 3.A Additional filtration map, M_add_(x,y), and emission map, M_emi_(x,y)

**Fig. 5:**
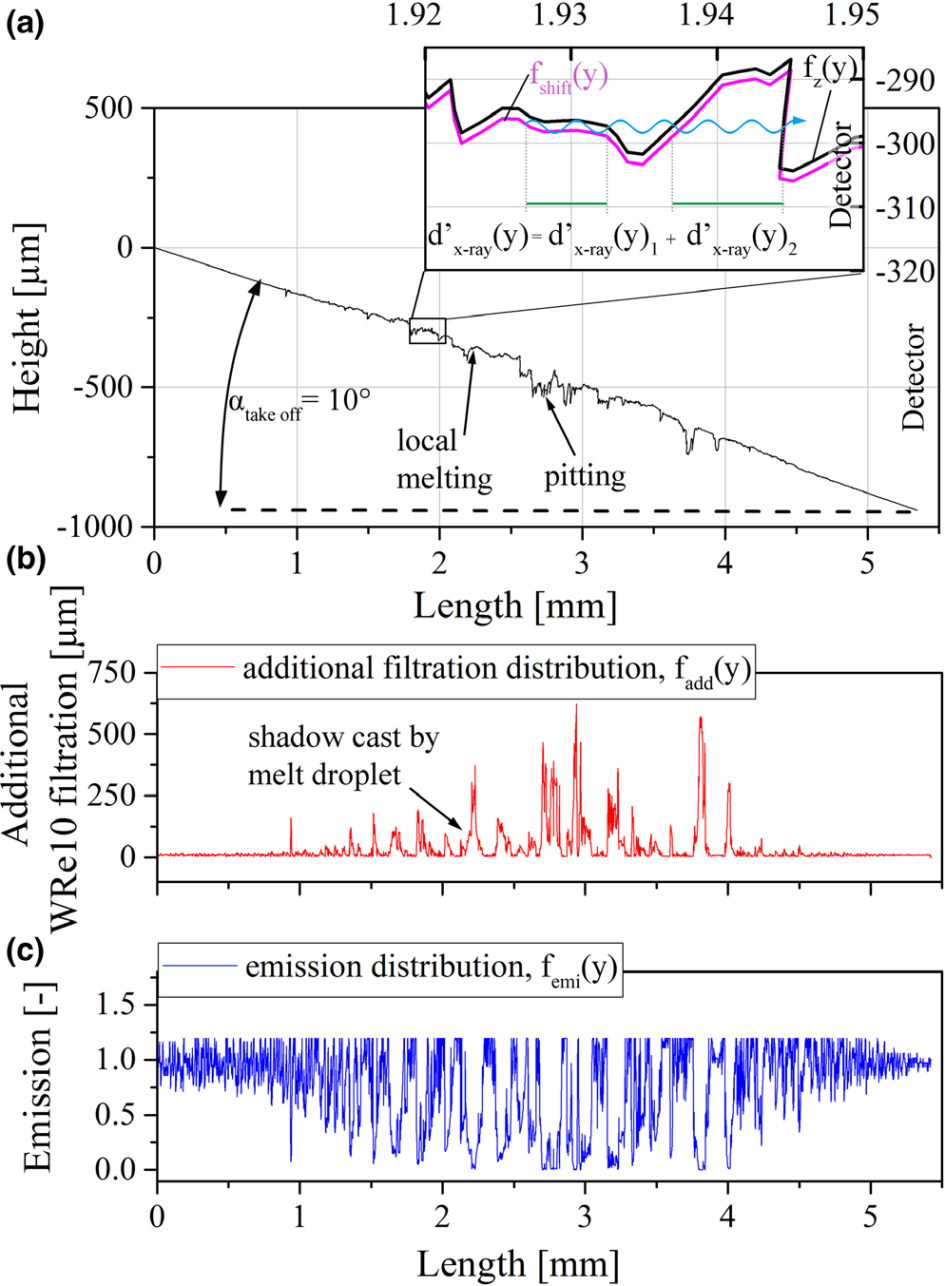
Calculation of f_add_(y) and f_emi_(y) represented on profile A; (a) calculation of d’_x‐ray_(y) for each pixel in the direction towards the detector; (b) calculated f_add_(y) for profile A; (c) calculated f_emi_(y) for profile A.

The first step of the geometrical model was to calculate the additional filtration map, M_add_(x,y), and the emission map M_emi_(x,y), starting from M_height_(x,y) of the LSCM data. Each map consisted of 5090 individual profiles in y‐direction which were extracted from the height images, processed and put back together. The y‐(detector) direction in relation to the sample surface is visualized in Fig. 3. The processing for profile A as marked by the dashed red line in Fig. 4(c) is visualized in Fig. 5 and is representative for all other profiles. Each extracted profile was tilted around the zero point according to α_take off_, Fig. 5(a). Afterwards, as illustrated in the insert, the profiles were shifted in –z direction according to d_e‐_ = 1.6 µm. For the calculation of the internal filtration distribution, d’_x‐ray_(y), the internal path in y‐direction between the shifted profile, f_shift_(y), and the original profile, f_z_(y), was calculated for each point of the profile. If several filtration events took place in the direction of the emitted x rays, the individual lengths, d’_x‐ray_(y)_i_, were added up to a total number:

(2) $$d_{x‐ray}^{\prime }\left(y\right)=\sum_{i=1}^{i=i_{\max}}d_{x‐ray}^{\prime }{\left(y\right)}_{i},$$

which is visualized in Fig. 5(a) in the insert. The minimal value of local WRe10 filtration was set to 5 µm which translates to a local take‐off angle off about 20° and no additional filtration events. When subtracting d_x‐ray_ = 9.07 µm at α_take off_ = 10° of a theoretically planar anode surface from d’_x‐ray_(y), one arrives at the additional filtration distribution:

(3) $$f_{\mathrm{add}}\left(y\right)=d_{\mathrm{xray}}^{\prime }\left(y\right)‐d_{x‐ray}.$$

f_add_(y) represents how much additional material the generated x rays have to penetrate in an aged anode compared with a planar anode surface till reaching the detector. For example, f_add_(y) for profile A can be found in Fig 5(b). In order to calculate the local emission distribution, f_emi_(y), which gives better insight on the relevance of the additional filtration, the relation between additional WRe10 filtration and x‐ray output reduction, f_red_(x), is necessary to be implemented. Therefore, f_emi_(y) is calculated by:

(4) $$f_{\mathrm{emi}}\left(y\right)=f_{\mathrm{red}}\left(f_{\mathrm{add}}\right).$$

Here, f_emi_(y) for profile A is shown in Fig. 5(c). It can be seen that additional filtration in the range of 200 µm, a value which is frequently surpassed in f_add_(y) of profile A, results in less than 10% x‐ray emission. If the M_add_(x,y) and M_emi_(x,y) are generated by calculating and adding up f_add_(y) and f_emi_(y) over the initial x‐resolution of the image.

**Fig. 6:**
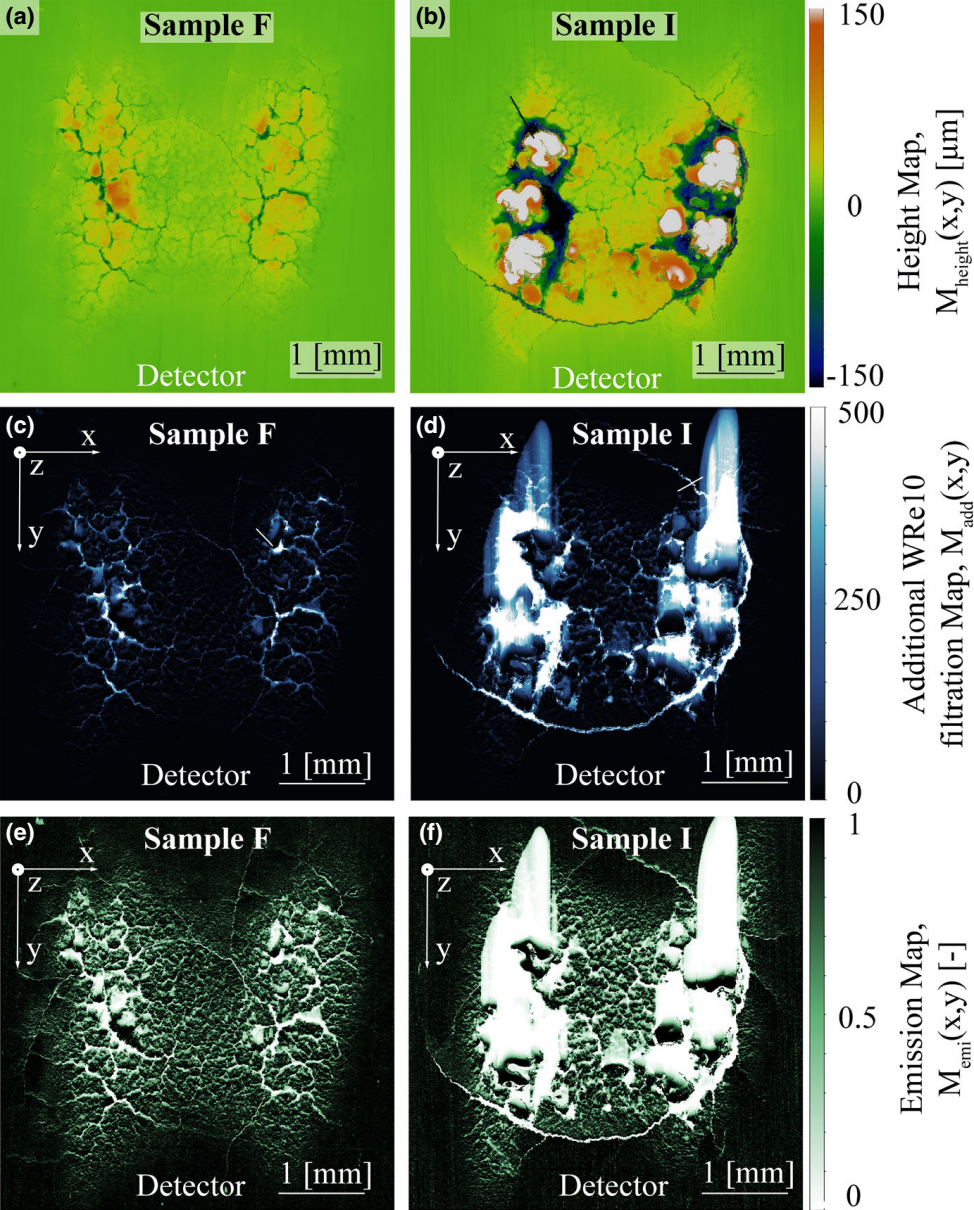
M_height_(x,y), (a) and (b), as well as M_add_(x,y), (c) and (d), for sample F and I. Large melt droplets cast extensive shadows in the direction away from the detector. M_emi_(x,y), (e) and (f), visualizes the impact of crack networks and surface roughening between the hot spots.

Figures 6(a) and 6(b) show M_height_(x,y) of sample F and I, in comparison to their calculated M_add_(x,y), see Figs. 6(c) and 6(d), as well as M_emi_(x,y), see Figs. 6(e) and 6(f). Sample F shows moderate surface damage, while sample I exhibits the most severe surface damage in terms of cracks, pitting, and local melting compared to all other samples tested.

Figures 6(c) and 6(d) visualize the additional WRe10 local path length the emitted x rays must penetrate to reach the detector due to the surface alteration. The direction of the detector is marked in the images. Large melt droplets on the surface due to local overheating on sample I cast shadows with high filtration in the detector turned away side. Pitting and cracks lead to the highest local values of additional filtration. However, especially at the heavily damaged sample I, less area is effected by pitting and cracks then by local melting. The plotted M_emi_(x,y) in Figs. 6(e) and 6(f) better visualizes the effect of only little additional WRe10 filtration on the x‐ray emission. The edges of the cast shadows become sharper and small cracks in between the hot spots lead to reduced emission. It can be seen the ground surface surrounding the focal spot is not leading to any significant reduction of emission.

### 3.B Local x‐ray output map, O’(x,y) and total x‐ray output reduction R_calculated_

Using Fig. 6 the influence of common surface features like cracks, melt droplets, pitting, or general surface roughening on the total x‐ray output reduction can be estimated. Looking at the characteristic hot spot appearance of the damaged samples, the electron input intensity is obviously not homogeneously distributed in the tests and, therefore, the calculated value from the geometric model would not be comparable to an air kerma measurement. In areas where the electron intensity is higher, and temperature and damage are increased (hot spots), there is a higher local x‐ray output to start with, which needs to be taken into account when calculating the total x‐ray output.

The local electron intensity function, f_e‐_(x,y), was modeled with two two‐dimensional Gaussian surface functions, one Gaussian function for each hot spot. The shape and size were based on the local x‐ray intensity distribution obtained by the image sensor. The positions of the two maxima were set to the hot spots in the LSCM image. Scalar multiplying M_emi_(x,y) with f_e‐_(x,y) yields the local x‐ray output map O’(x,y):

(5) $$O^{\prime }\left(x,y\right)=M_{\mathrm{emi}}\left(x,y\right)\cdot f_{e‐}\left(x,y\right).$$

**Fig. 7:**
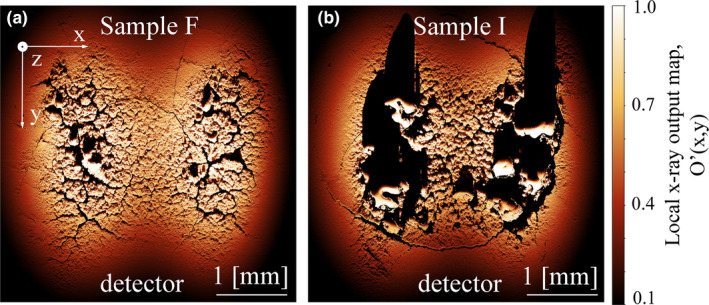
Local x‐ray output map O’(x,y) of sample F (a) and sample I (b).

Figure 7 shows the calculated O’(x,y) for samples F and I. The values range from 1, a point with maximum emission and maximum electron intensity to 0, a point with either no electron intensity or high additional filtration. In general, the images look comparable to M_emi_(x,y) in Fig. 6 and different features still throw shadows according to their height and orientation toward the detector. The main difference is that if features are located in areas with low electron intensity, their impact on the total x‐ray output is of little to no importance for the air kerma measurement. When now summarizing O’(x,y) over the x‐ and y‐resolution of one image, 5090 × 5090 pixels, the total x‐ray output O’_total_ can be calculated according to:

(6) $$O_{\mathrm{total}}^{\prime }=\sum_{x=0}^{5090}\sum_{y=0}^{5090}O^{\prime }\left(x,y\right).$$

The relative difference between O’_total_ of a sample with surface alteration and the calculated total x‐ray output of a ground sample, O_total_, yields the total x‐ray output reduction of the sample:

(7) $$R_{\mathrm{calculated}}=1‐\frac{O_{\mathrm{total}}^{\prime }}{O_{\mathrm{total}}}.$$

R_calculated_ now can be directly compared with the air kerma measurement R_measured_ at the test system. For samples F and I their respective R_calculated_ is 16% and 38%.

**Fig. 8:**
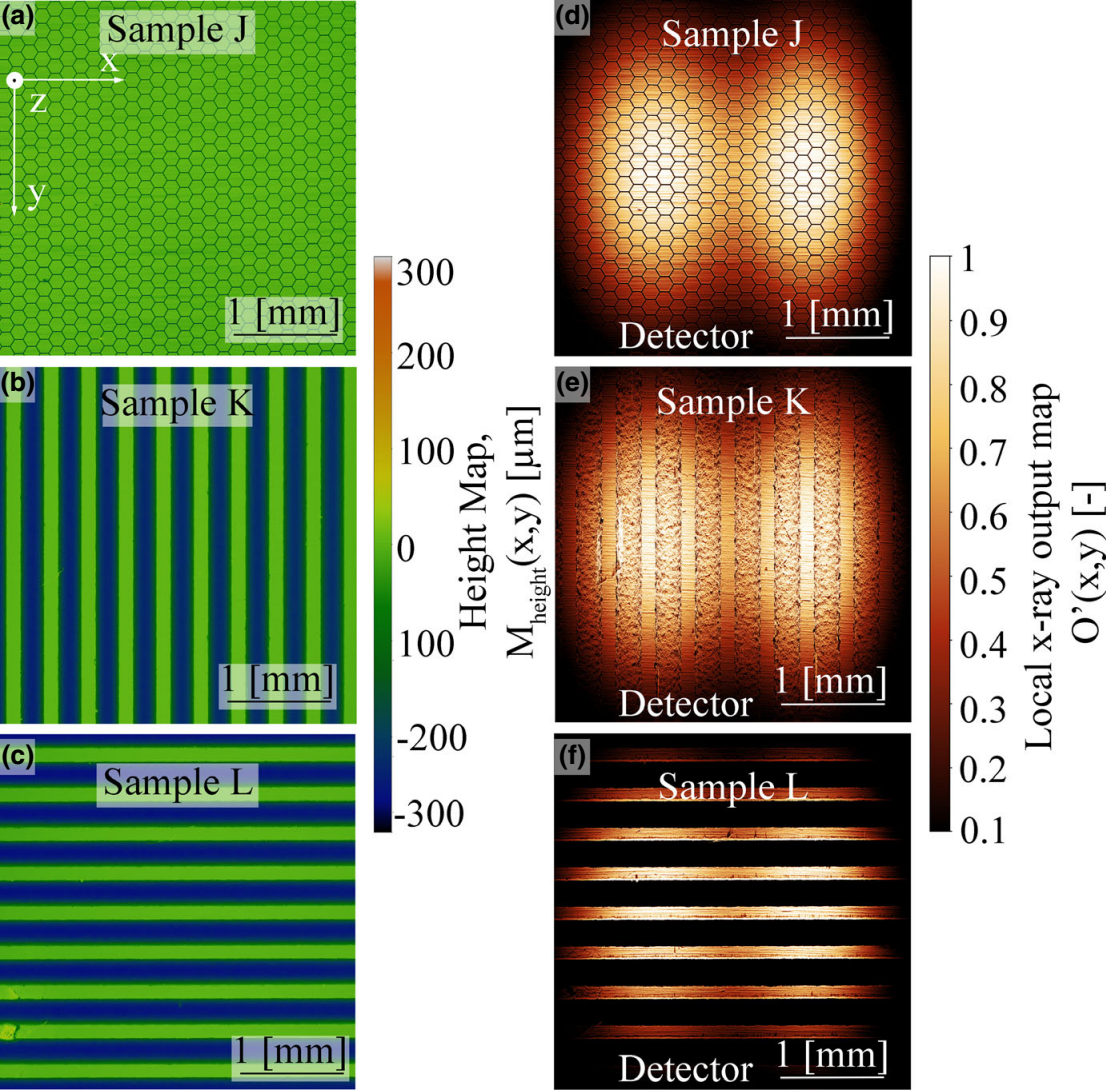
M_height_(x,y) and O’(x,y) for the surface structured samples: (a)–(c) M_height_(x,y) of samples J‐L; (d)–(g) O’(x,y) for samples J‐L.

Results from the surface analysis for samples J–L (with surface structuring) are shown in Fig. 8. Sample J resembles a honeycomb‐like surface structure with a characteristic structure size of 250 μm and a maximum groove depth of about 50 μm. Samples K and L both have a grooved surface with an average depth of 250 μm for both samples; the area taken away by the grooves was 60% for sample K and 65% for sample L. For sample K, the grooves were orientated toward the detector, in the y‐direction, and for sample L they were perpendicular to it, in the x‐direction. As shown in Fig. 8 for samples J, K, and L: if a sample has a homogeneous surface structuring with its characteristic feature size being significantly smaller than the focal spot size, an inhomogeneous intensity distribution should have only little to no impact on R_measured_.

## 4 DISCUSSION

**Fig. 9:**
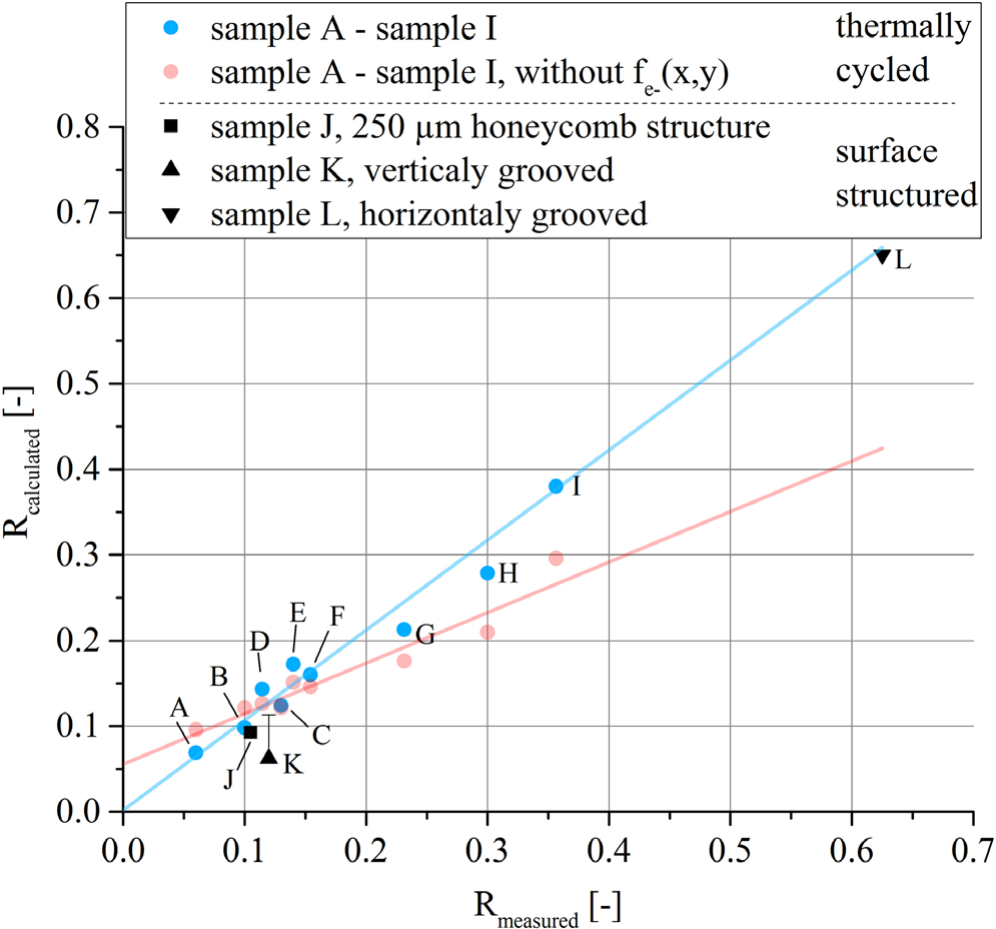
Comparison of R_calculated_ with R_measured_ at U_tube_ = 100 kV, α_take off_ = 10° and 2 mm Al filtration.

Figure 9 shows the comparison of R_calculated_ and R_measured_ at α_take off_ =10° and U_tube_ = 100 kV. All blue dots (online version only) represent samples damaged by thermal cycling and tested for air kerma reduction with the typical surface appearance like crack formation, pitting and formation of melt droplets. The strong directly proportional relation between R_measured_, and R_calculated_ shows that even with the applied simplifications, the additional internal filtration due to geometric surface modification is by far the most important factor regarding the measured x‐ray output reduction. The red line and the red dots (online version only) in Fig. 9 show R_calculated_ without applying the inhomogeneous electron intensity distribution by setting f_e‐_(x,y) = 1. Although R_calculated_ still shows a linear correlation with R_measured_, the x‐ray output reduction is underestimated for severely damaged samples and overestimated for lesser damaged samples. The calculated values of samples with artificial surface modifications, J and L, are in good agreement with the measurements when applying the inhomogeneous electron intensity distribution, Fig. 9. For sample K, the model underestimates R_measured_ by a factor of 1.9. The reason for this is the high sensitivity to a slight misalignment for this type of sample. If the sample is rotated around the z‐axis in the model by just 3°, R_calculated_ would reach 11%, which is indicated in the scatter bar in Fig. 9 and would be in good agreement with R_measured_. For sample L the measured reduction in air kerma rate matches the area taken away by the grooves. Even though sample K does not follow the model to the last detail, the large difference in R_measured_ between sample K and sample L points out how crucial is the alignment of surface features in relation to the detector. This shows that if the effect of an inhomogeneous electron intensity distribution is canceled out, the geometric model calculates similar reasonable values for the x‐ray output. Furthermore, it shows that for nonconventional surface alterations the x‐ray output reduction can still be calculated by modeling the increased local internal absorption. Surface structuring of rotating anodes is a topic which can be found in various patents aiming for increasing thermal emission,^23^ reducing thermal stresses,^24^, ^25^, ^26^, ^27^ or introducing preferential cracks paths.^28^ The orientated surface structuring of sample K increases the surface area by 150% while simultaneously showing only 5% of initial x‐ray output reduction.

The increased internal absorption attenuates low energy x rays more strongly than high energy x rays. Consequently, a high level of added filtration reduces the effect of the surface roughening on the total x‐ray output.^4^ The measured total x‐ray output reduction is originating from a relatively small area with high filtration rather than a larger area having intermediate filtration. Consequently, only small changes of the spectra and the measured half value layer (HVL) can be expected. The tested samples in this work only showed minimal HVL increases from a value after conditioning of 3.9 ± 0.04 mm Al to a maximum at heavily damaged samples of 4.1 ± 0.04 mm Al. The measured HVL values are in good agreement with the expected HVL values at U_tube_ = 100 kV, α_take off_ = 10°, the 2.5 mm borosilicate glass window and the added filtration of 2 mm Al used in the experiments. Other authors found comparable results in the sense that increased tube aging rather manifests in decreasing x‐ray output when investigating low α_take off_ and high U_tube_ than in changed HVL.^1^, ^2^, ^11^

**Fig. 10:**
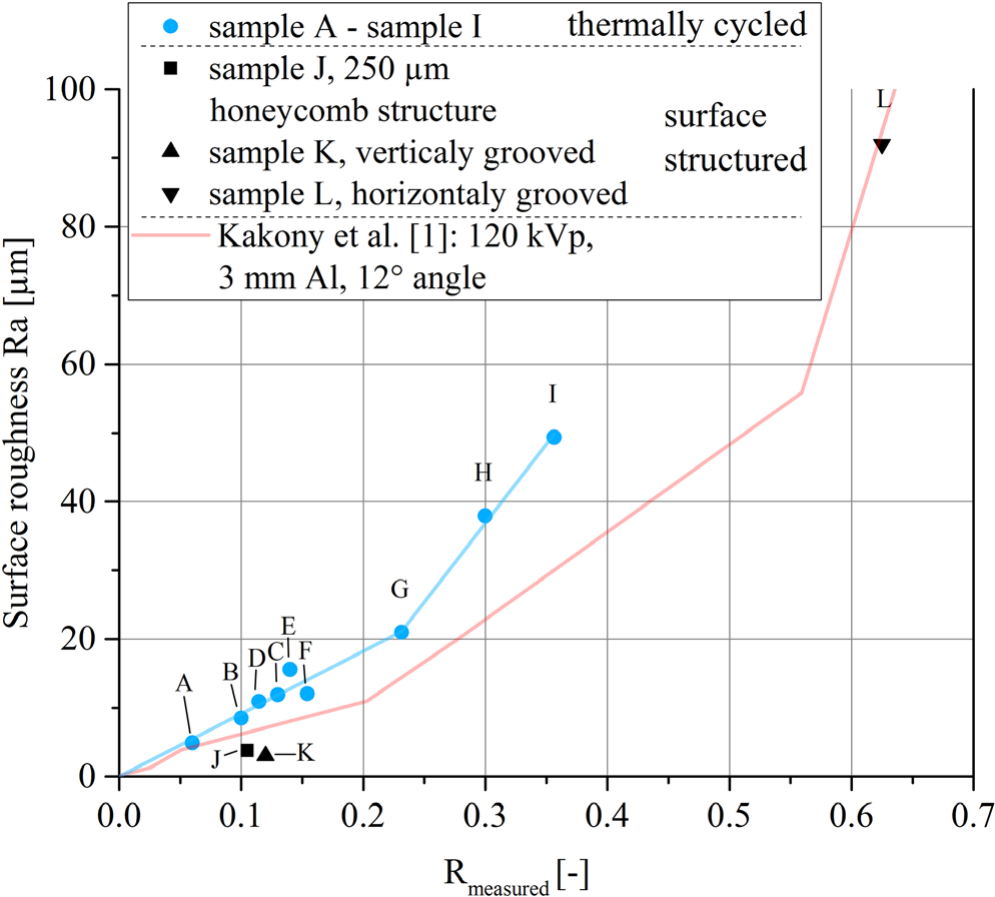
Comparison of R_measured_ with surface roughness R_a_ at U_tube_ = 100 kV, α_take off_ = 10° and 2 mm Al filtration.

The roughness, that is, R_a_, might be a value that is easier and faster to obtain than a fully three‐dimensional dataset of the surface. Figure 10 shows R_measured_ plotted against the measured R_a_ values. Up to R_a_ = 20 µm a roughly linear relation can be found within the investigated samples A to G. At higher surface roughness the impact of increased surface roughness on the total x‐ray output reduction declines. Shadows cast by melt droplets are beginning to overlap as well as the depth of pits and cracks has less effect on the x‐ray output than their projected area. Kákonyi et al.^1^ investigated a large amount of real and modeled surfaces and calculated their relative x‐ray intensity output by Monte Carlo simulation. The most comparable situation to the tests in this work, which is U_tube_ = 120 kV, 3 mm Al, α_take off_ = 12°, is visualized for comparison in Fig. 10. They clearly pointed out that surface roughness R_a_ by itself is not sufficient to describe the x‐ray intensity output reduction in general, since besides α_take off_ and U_tube_, the exact surface geometry has a strong impact. When these authors investigated similar types of surface profiles in their work, the surface roughness R_a_ turns out to scale close to linear with their calculated intensity reduction up to R_a_ = 60 µm. Beyond R_a_ = 60 µm, the impact of the surface roughness on the calculated intensity reduction decreases. In theory, the reduced α_take off_ of 10° used in the current investigation should lead to a shift of this transition to lower R_a_ values as it is observed in Fig. 10. The surface roughness of recovered rotating anodes after failure rarely exceeds R_a_/S_a_ = 10 µm or R_z_ = 45 µm.^1^, ^3^, ^7^, ^26^ For example, the surfaces shown in Figs. 1(a) and 1(b) calculate to R_a_ = 1.8 µm, R_z_ = 15 µm and R_a_ = 4.5 µm, R_z_ = 32 µm. The results show that for rotating anodes, which have similar surface features and are operated by similar parameters, the surface roughness is an acceptable rating parameter for absolute x‐ray output in the technical relevant field of U_tube_ = 100 kV, 2 mm Al and α_take off_ = 10°. Highly damaged rotating anodes surfaces often show severe amounts of local melting. In such case, the x‐ray output reduction could be overestimated with R_a_ roughness measurements due to the overlapping of the feature shadows.

In order to translate the shown model to other clinically relevant scenarios, detailed knowledge of the individual electron intensity distribution and high resolution surface data are necessary.^29^, ^30^, ^31^, ^32^ Furthermore, two major simplifications were applied in this work, that is, taking only the average depth of electron penetration into account and calculating the x‐ray output strictly in y‐direction. The major simplification of taking only the average depth of electron penetration into account resembles the real case to an acceptable degree within the applied boundaries investigated here. When considering the insert in Fig. 5(a), which represents f_shift_(y) and d_e‐_ with the x‐ and y‐axis in a one to one scale, it seems reasonable that a subdivision into several depths will not make a large difference to the calculated internal pathways and, consequently, to R_calculated_. In the investigated field, only a few micrometers of additional W are enough to strongly attenuate the x‐ray emission. Regarding stationary anodes, the strict calculation in purely the y‐direction could also have an impact when only little collimation is applied or when the distance between the x‐ray source and the detector is small. This situation could be improved by calculating R_calculated_ in a more realistic fan or applying higher collimation when testing. Both simplifications, and consequently the geometric model, should be reasonably applicable to other scenarios, for example, high‐performance rotating anodes, as long as the anode exhibits at least a medium degree of surface roughening. For anodes with slightly roughened surfaces the minor changes of the x‐ray output will be more strongly influenced by the local take off angle than by the shadows cast by the different surface features. For such cases a more detailed treatment of the electron depth‐ and energy distribution has to be implemented into the model.^12^, ^16^ However, since differences remain in the surface appearance of rotating anodes and the stationary anodes shown here, further work on rotating anodes should be conducted to show the effect at specific clinically relevant scenarios.

In many cases, a loss of x‐ray output can be compensated by longer exposure times or higher electrical power input during exposure. However, certain techniques do not allow for longer exposure times and the applied electrical power input might already be driving the materials to their limits. Increased electrical power input would then damage the surface of the anode even further during operation and ultimately lead to a negative feedback loop, at the end of which an unusable x‐ray tube is obtained.

## 5 CONCLUSIONS

The geometric model established in this paper predicts a close linear correlation with the measured air kerma reduction with less than 10% average error. The alteration of the anode surface resulting from thermal cycling during operation was identified as the main contributor regarding x‐ray tube aging. It was shown that the model enables the estimation of the impact of any given surface geometry on the x‐ray output for U_tube_ at 100 kV, α_take off_ =10° and the applied 2 mm Al filtration. Characteristic surface features like cracks, pitting, and melt droplets influence the total x‐ray output based on their size and orientation toward the detector. For surfaces showing comparable characteristic features as in rotating anodes, surface roughening R_a_ proved to be an acceptable rating parameter for estimating differences in x‐ray output reduction in close boundaries. Orientated surface structuring enabled the increase in surface area by 150%, while leading to only minimal initial x‐ray output reduction of 5% or less.
